# Small nucleolar RNA 113–1 suppresses tumorigenesis in hepatocellular carcinoma

**DOI:** 10.1186/1476-4598-13-216

**Published:** 2014-09-14

**Authors:** Gang Xu, Fang Yang, Cui-Ling Ding, Lan-Juan Zhao, Hao Ren, Ping Zhao, Wen Wang, Zhong-Tian Qi

**Affiliations:** Department of Microbiology, Shanghai Key Laboratory of Medical Biodefense, Second Military Medical University, 800 XiangYin RD, Shanghai, 200433 China; Department of Hepatobiliary Surgery, Fuzhou General Hospital of Nanjing Military Area Command, Fuzhou, 350025 China

## Abstract

**Background:**

Emerging evidence suggests that small nucleolar RNAs (snoRNAs) are involved in tumorigenesis. The roles of small nucleolar RNA 113–1 (SNORD113-1) on the development of hepatocellular carcinoma (HCC) remain unknown.

**Methods:**

The expression of SNORD113-1 was measured in 112 HCC tumor tissues using quantitative RT-PCR and compared with expression levels from with paired non-tumor tissues. The effects of SNORD113-1 on HCC tumorigenesis were investigated in HepG2 and Huh7 cells as well as a xenograft nude mouse model. CpG methylation within the promoter region of the SNORD113-1 gene was identified using Sodium bisulfite sequencing. Cancer pathway reporter investigate the mechanism by which SNORD113-1 suppressed tumorigenesis.

**Results:**

SNORD113-1 expression was significantly downregulated in HCC tumors compared with adjacent non-tumor tissues, and downregulation of SNORD113-1 in HCC tumors was significantly associated with worse survival of patients. In addition, CpG methylation at the promoter region of the SNORD113-1 gene was higher in HCC tumors than adjacent non-tumor tissues. Functionally, SNORD113-1 suppressed cancer cell growth in HepG2 and Huh7 cells and in a xenograft nude mouse model. Furthermore, SNORD113-1 inactivated the phosphorylation of ERK1/2 and SMAD2/3 in MAPK/ERK and TGF-β pathways.

**Conclusions:**

SNORD113-1 functions as a tumor suppressor role in HCC and may be important as a potential diagnostic and therapeutic target for HCC.

**Electronic supplementary material:**

The online version of this article (doi:10.1186/1476-4598-13-216) contains supplementary material, which is available to authorized users.

## Background

Hepatocellular carcinoma (HCC) is the third leading cause of cancer deaths [1]. The development and progression of HCC occurs as a typical multistage disease, in which a number of genes related to cellular processes such as cell cycle control, cell growth, apoptosis and cell migration, are deregulated [2]. Recently, an increasing number of studies investigating the role of non-coding RNAs (ncRNAs) in the pathology of HCC have been reported, including microRNAs (miRNAs), long non-coding RNA (lncRNAs) and small nucleolar RNAs (snoRNAs) [3–5].

SnoRNAs belong to a group of ncRNA molecules of that are in the range of 60–300 nucleotides in length. This group of ncRNAs is predominantly found in the nucleolus and functions to guide RNAs for post-transcriptional modification of ribosomal RNAs and some spliceosomal RNAs [6]. Recent research has suggested that malfunctioning snoRNAs may have roles in the development and progression of human malignancy. For instance, SNORA42 has been shown to act as an oncogene in lung tumorigenesis [7], SNORD33, SNORD66 and SNORD76 are potential markers for non-small-cell lung cancer [8], and HBII-239 snoRNA may have diagnostic and prognostic significance for peripheral T-cell lymphoma [9]. Accumulating evidence suggests that snoRNAs may be actively involved in carcinogenesis and play diverse roles in tumor biology.

In the present study, we demonstrated that small nucleolar RNA 113-1(SNORD113-1) was significantly downregulated in HCC tissues as compared with adjacent non-tumor tissues, and this downregulation of SNORD113-1 was associated with decreased survival of HCC patients. Furthermore, we found that SNORD113-1 was regulated by CpG island methylation of the putative promoter region. Functional analyses indicated SNORD113-1 inhibited both cell growth and tumorigenicity of HCC cells, possible through effects on MAPK/ERK and TGF-β pathways.

## Results

### Differential expression of mRNAs in HCC

To identify novel differential gene expression in HCC tissues, Human Transcriptome Arrays were performed on 3 pairs of HCC tumors and adjacent non-tumor tissues (Table 1A). In total, 3233 differentially expressed mRNAs were identified (fold change ≤ -0.5 or ≥ +2, P-value ≤ 0.05), including 2065 upregulated and 1168 downregulated mRNAs. 29 upregulated mRNAs and 35 downregulated mRNAs showed greater than 10-fold changes compared to non-tumor tissues (Table 2). The four most upregulated genes were pepsinogen C (PGC), alpha fetoprotein (AFP), aldoketo reductase family 1 member B10 (AKR1B10) and glypican 3 (GPC3), which showed greater than 30-fold higher expression in HCC tumors than adjacent non-tumor tissues. In contrast, the five genes that demonstrated the most significant downregulation were tyrosine aminotransferase (TAT), Jun dimerization protein 2 (JDP2), hydroxysteroid (17-beta) dehydrogenase 13 (HSD17B13), cytochrome P450 family 2 subfamily B polypeptide 6 (CYP2B6), and phosphoenolpyruvate carboxykinase 1 (PCK1), which showed greater than 30-fold lower expression in HCC tumors than adjacent non-tumor tissues. Interestingly, four C/D box small nucleolar RNAs, including SNORD113-1, SNORD114-1, SNORD113-6 and SNORD114-17, were significantly downregulated in tumor tissues compared to normal liver tissue.

**Table 1:** **The data of patients**

A				
Patients	Age	Tumor size	Tumor grade	TNM stage
P1	58	12	G3	III
P2	63	10.5	G3	III
P3	66	9.5	G3	II
**B**				
**Variable (n=112)**				**Value**
**Age (years)**				53.5±11.4
**Gender (male)**				41(73.2%)
Tumor size* (cm)				7.62
**Tumor grade**				
Well-differentiated (G1-2)				32
Moderately-differentiated (G3)				76
Poorly-differentiated (G4)				4
TNM stage**				
I				20
II				54
III				32
IV				6

^*^Diameter of the biggest nodule.

^**^TNM: tumor-node-metastasis.

**Table 2:** **Deregulated mRNAs in Hepatitis B virus-associated HCC**

Symbol	Description	Fold change	***p***-value
**Up-regulated mRNAs**			
PGC	progastricsin (pepsinogen C)	47.64004927	0.0023
AFP	alpha-fetoprotein	44.39471392	0.0034
AKR1B10	aldo-keto reductase family 1, member B10 (aldose reductase)	34.90323855	0.0031
GPC3	glypican 3	33.3617948	0.0025
REG3A	regenerating islet-derived 3 alpha	29.11429945	0.0016
SPP1	secreted phosphoprotein 1 (osteopontin, early T-lymphocyte activation 1)	23.96828868	0.0031
SQLE	squalene epoxidase	23.32641995	0.0035
FADS2	fatty acid desaturase 2	20.89067206	0.0015
CDR1	cerebellar degeneration-related protein 1, 34kDa	18.83885007	0.0034
ACSL4	acyl-CoA synthetase long-chain family member 4	17.80369764	0.0051
Q9BT26_HUMAN	MGC10981 protein. [Source:Uniprot/SPTREMBL; Acc:Q9BT26]	16.69515983	0.0016
MEP1A	meprin A, alpha (PABA peptide hydrolase)	16.42375379	0.0023
TOP2A	topoisomerase (DNA) II alpha 170kDa	16.41288559	0.0031
MKI67	antigen identified by monoclonal antibody Ki-67	15.96034575	0.0035
NQO1	NAD(P)H dehydrogenase, quinone 1	15.93951691	0.0042
CENPF	centromere protein F, 350/400ka (mitosin)	14.37592617	0.0028
ANLN	anillin, actin binding protein	14.34102265	0.0018
Q6ZN80_HUMAN	CDNA FLJ16351 fis, clone TESTI2039060, moderately similar to Maltase- glucoamylase, intestinal. [Source: Uniprot/SPTREMBL; Acc:Q6ZN80]	14.12849608	0.0037
ASPM	asp (abnormal spindle) homolog, microcephaly associated (Drosophila)	13.47005653	0.0031
PROM1	prominin 1	12.79763216	0.0029
SPINK1	serine peptidase inhibitor, Kazal type 1	12.7974104	0.0051
Q6ZUK9_HUMAN	CDNA FLJ43606 fis, clone SPLEN2009548 (Hypothetical LOC613266). [Source: Uniprot/SPTREMBL; Acc:Q6ZUK9]	12.61622159	0.0037
SULT1C2	sulfotransferase family, cytosolic, 1C, member 2	12.22079301	0.0029
LYZ	lysozyme (renal amyloidosis)	11.95607544	0.0016
LGR5	leucine-rich repeat-containing G protein-coupled receptor 5	11.46664156	0.0028
HIST2H4A	histone cluster 2, H4a	10.97942046	0.0034
FMO1	flavin containing monooxygenase 1	10.69015599	0.0009
NUSAP1	nucleolar and spindle associated protein 1	10.56415889	0.0038
BPIL1	bactericidal/permeability-increasing protein-like 1	10.11522846	0.0051
***Down-regulated miRNAs***			
TAT	tyrosine aminotransferase	0.016479668	0.0037
JDP2	Jun dimerization protein 2	0.017312537	0.0027
HSD17B13	hydroxysteroid (17-beta) dehydrogenase 13	0.018812312	0.0018
CYP2B6	cytochrome P450, family 2, subfamily B, polypeptide 6	0.025612431	0.0031
PCK1	phosphoenolpyruvate carboxykinase 1 (soluble)	0.029105346	0.0023
NR_003229.1	**small nucleolar RNA, C/D box 113–1 (SNORD113-1) on chromosome 14 [Source: RefSeq_dna; Acc:NR_003229]**	0.040807072	0.0028
GLYAT	glycine-N-acyltransferase	0.041146571	0.0034
NR_001278.1	cytochrome P450, family 2, subfamily B, polypeptide 7 pseudogene 1 (CYP2B7P1) on chromosome 19 [Source: RefSeq_dna; Acc:NR_001278]	0.046105574	0.0037
HSD11B1	hydroxysteroid (11-beta) dehydrogenase 1	0.047541954	0.0046
ABCA8	ATP-binding cassette, sub-family A (ABC1), member 8	0.048856354	0.0008
SDS	serine dehydratase	0.049689196	0.0035
CYP1A2	cytochrome P450, family 1, subfamily A, polypeptide 2	0.052959819	0.0019
MFSD2	major facilitator superfamily domain containing 2	0.053182596	0.0016
GYS2	glycogen synthase 2 (liver)	0.054336295	0.0031
SLC22A1	solute carrier family 22 (organic cation transporter), member 1	0.059035231	0.0029
ADH4	alcohol dehydrogenase 4 (class II), pi polypeptide	0.061977699	0.0046
NR_003193.1	**small nucleolar RNA, C/D box 114–1 (SNORD114-1) on chromosome 14 [Source: RefSeq_dna; Acc: NR_003193]**	0.064302828	0.0027
BCO2	beta-carotene oxygenase 2	0.068982298	0.0031
PZP	pregnancy-zone protein	0.068984067	0.0061
HPD	4-hydroxyphenylpyruvate dioxygenase	0.069601683	0.0043
MT1G	metallothionein 1G	0.071319826	0.0037
CRHBP	corticotropin releasing hormone binding protein	0.073777924	0.0031
MT1M	metallothionein 1M	0.076630206	0.0026
FOS	v-fos FBJ murine osteosarcoma viral oncogene homolog	0.078040509	0.0052
COLEC10	collectin sub-family member 10 (C-type lectin)	0.085870417	0.0048
NR_003234.1	**small nucleolar RNA, C/D box 113–6 (SNORD113-6) on chromosome 14 [Source: RefSeq_dna; Acc: NR_003234]**	0.086148113	0.0037
SLC10A1	solute carrier family 10 (sodium/bile acid cotransporter family), member 1	0.086419698	0.0034
CYP8B1	cytochrome P450, family 8, subfamily B, polypeptide 1	0.088848979	0.0016
CNDP1	carnosine dipeptidase 1 (metallopeptidase M20 family)	0.089111411	0.0018
FOSB	FBJ murine osteosarcoma viral oncogene homolog B	0.092529294	0.0027
MT1P2	metallothionein 1 pseudogene 2	0.092597882	0.0034
C7	complement component 7	0.093307121	0.0036
NR_003210.1	**small nucleolar RNA, C/D box 114–17 (SNORD114-17) on chromosome 14 [Source: RefSeq_dna; Acc: NR_003210]**	0.094172719	0.0043
CYP3A4	cytochrome P450, family 3, subfamily A, polypeptide 4	0.096675826	0.0019
XDH	xanthine dehydrogenase	0.099523084	0.0022

### Downregulation of SNORD113-1 was associated with aggressive biological behavior of HCCs

**Figure 1:**
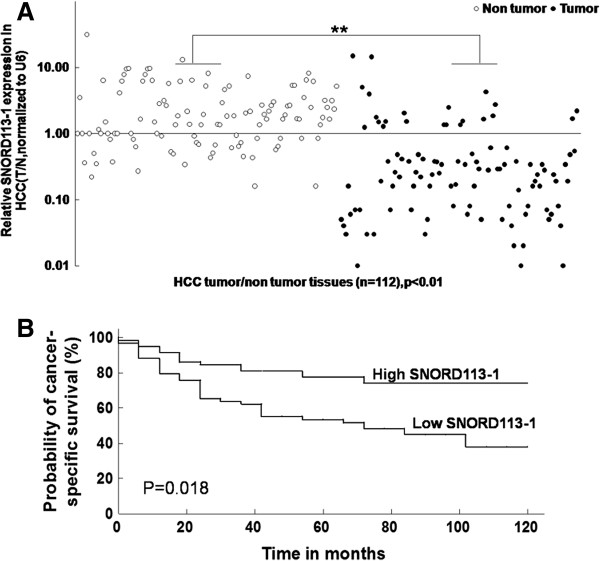
**SNORD113-1 was downregulated in HCC tumor tissues. A**. Downregulated expression of SNORD113-1 in HCC tumor tissues compared with adjacent non-tumor tissues (n = 112,*p < 0.05, **p < 0.01). **B**. Probability of cancer-specific survival determined using levels of SNORD113-1 expression in HCCs.

To validate the array data, qRT-PCR was performed on RNA extracted from 112 pairs of HCC and non-tumor tissues. All of the four snoRNAs identified in our microarray analyses demonstrated downregulation in greater than 50% HCC tumors (data not shown), of which SNORD113-1 was downregulated in 77.6% (87/112) of HCC samples (Figure 1A).Furthermore, patients whose primary tumors did not demonstrate significant downregulation of SNORD113-1 had a trend of increased relapse-free survival compared with those patients whose primary tumors demonstrated SNORD113-1 downregulation (Figure 1B), independent of the tumor stage or size. The mean relapse-free survival in patients with low SNORD113-1 expression (n = 87) was 68.4 months, whereas the mean relapse-free survival in those with high SNORD113-1 expression (n = 25) was 99.8 months. No statistically significant correlations were observed between SNORD113-1 expression and other clinically pathological features (age, sex, HBV viral load, HBsAg or HBeAg) (data not shown).

### CpG hypermethylation downregulated SNORD113-1 expression in HCC

**Figure 2:**
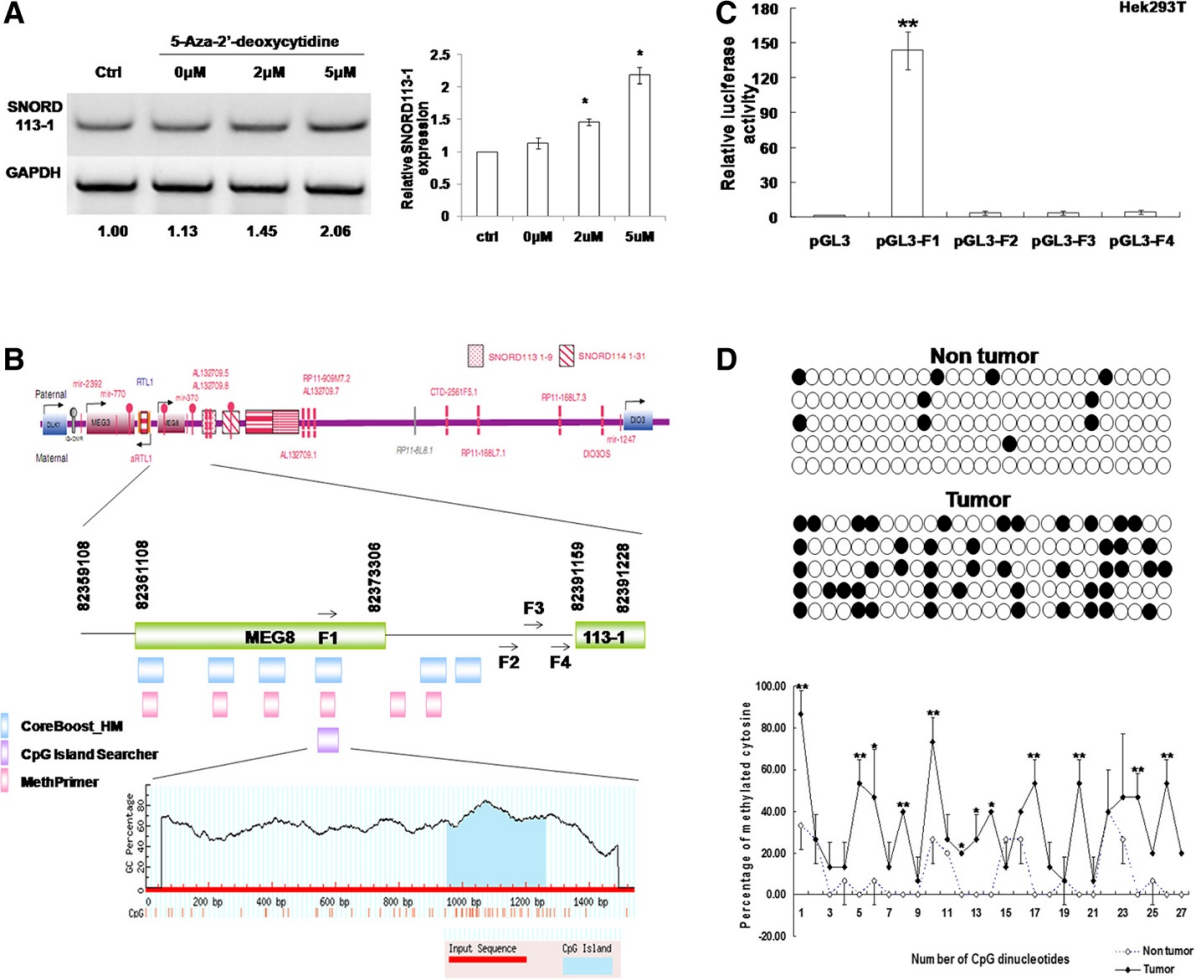
**Hypermethylation of CpGs mediated decreased SNORD113-1 expression. A**. Demethylation following treatment with 5-aza-2′-deoxycytidine induced SNORD113-1 upregulation. Left panel: a representative result; Right panel: the results from three experiments. **B**. Upper panel: a schematic of the Dlk1-Dio3 region in 14q32 [10]; Lower panel: the CpG island enriched sites in the upstream region of SNORD113-1 were analyzed using CpG island searcher, CoreBoost_HM and MethPrimer. **C**. Strong promoter activity of fragment 1 (F1) was demonstrated using luciferase assays. **D**. Bisulfite sequencing of the putative promoter region. Left panel, each line represents one PCR product, and five PCR products are shown for each sample. ●, methylated CpGs; ○, unmethylated CpGs. Right panel, the methylation status of each CpG in this region was quantified using the percentage of methylated CpGs among all PCR products. *p < 0.05, **p < 0.01.

The methylation inhibitor, 5-Ad, was used to demethylate genomic DNA. As shown in Figure 2A, SNORD113-1 expression levels were increased 2.06-fold in HepG2 cells following treatment with 5 μmol/L 5-Ad for 48 h compared with vehicle-treated cells.

The Dlk1-Dio3 genomic region contains the paternally expressed genes Dlk1, Rtl1, and Dio3, and the maternally expressed genes MEG3 and MEG8 (Figure 2B, upper panel) [10]. This region also hosts 54 miRNAs and many snoRNAs, in particular 1, 9, and 31 paralogous copies of SNORD112, SNORD113 and SNORD114, respectively (Figure 2B, upper panel) [10]. To explore the relationship between promoter methylation and SNORD113-1 gene downregulation, we utilized CpG island prediction software to analyze the promoter region of SNORD113-1. As shown in Figure 2B and Additional file 1: Table S2, fragment 1 (F1, 82370922 ~ 82371234 in chromosome 14, NC_000014.8) was rich in CpG dinucleotides and predicted as a putative promoter region by three software programs.To test whether fragments 1–4 were functionally important, promoter assays were performed using reporter plasmids containing these four regions in Hek293T cells. In transient transfection experiments, F1 displayed 143.5-fold higher promoter activity than those of the pGL3-basic lacking eukaryotic promoter and enhancer control plasmids (Figure 2C), respectively. These data clearly indicated that F1 contained functionally important sequences for gene expression.To examine the methylation pattern, F1, containing a total of 27 CpG dinucleotides, was amplified from genomic DNA isolated from HCC and non-tumor tissues treated with either sodium bisulfite or vehicle. Figure 2D shows a representative methylation pattern of the 27 CpGs putative promoter region. In non-tumor tissues, the majority of CpGs were unmethylated, whereas in the tumor tissues, most of the CpGs were methylated (Figure 2D, left panel). The methylation status of each CpG was quantified using the percentage of methylated CpGs among all PCR products analyzed. 44.4% (12/27) CpGs were hypermethylated in tumors compared with normal pituitaries (Figure 2D, right panel). Thus, methylation in the putative promoter region of SNORD113-1 gene was higher in HCC tumor tissues than in those from adjacent non-tumor tissues.

### SNORD113-1 suppressed HCC tumorigenesis *in vitro*and *in vivo*

**Figure 3:**
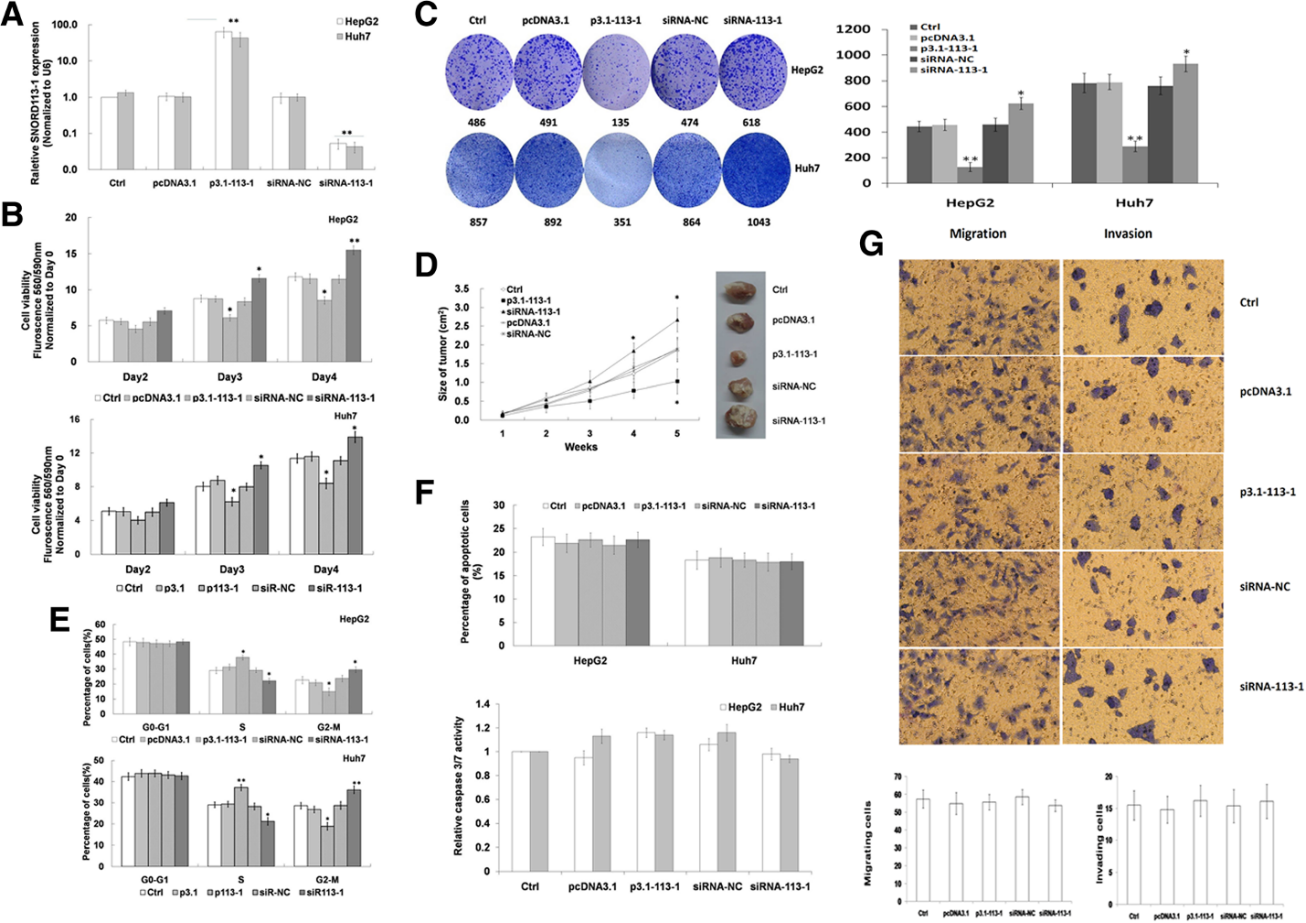
**SNORD113-1 inhibited colony formation in culture and tumorigenesis. A**. The expression of SNORD113-1 in HepG2 (□) and Huh7 (■) cells with or without infection of p3.1-SNORD113-1, SNORD113-1 siRNA or NC duplex. **B**. The effects of SNORD113-1 on cell viability in HepG2 cells. **C**. The effects of SNORD113-1 on colony formation in HepG2 cells. Left panel: the representative result of a colony formation assay; Right panel: the mean value from all three independent experiments determining colony formation. **D**. The effects of SNORD113-1 on xenograft tumor growth in nude mice. **E**. The effects of SNORD113-1 on cell cycle in HepG2 cells. **F**. The effects of SNORD113-1 on apoptosis in HepG2 cells. Upper panel: the results of apoptosis analysis measured with flow cytometry; Lower panel: the results of apoptosis analysis determined using caspase3/7 activity measurements. **G**. The effects of SNORD113-1 on cell migration and invasion in HCC cell lines. Upper panel: the representative result of cell migration and invasion; Lower panel: the mean value from at least three independent experiments of cell migration and invasion. *p < 0.05, **p < 0.01.

The significant downregulation of SNORD113-1 expression in HCC tissues suggested possible biological significance in tumorigenesis. First, we evaluated the effects of SNORD113-1 on cell growth in HepG2 and Huh7 cells transfected with either p3.1-SNORD113-1 or SNORD113-1 siRNA. Measured using qRT-PCR, the expression of SNORD113-1 was 57.4-fold (HepG2) and 63.5-fold (Huh7) increased in cells transfected with p3.1-SNORD113-1, but 32.2-fold (HepG2) and 23.4-fold (Huh7) deceased in cells transfected with 20nM SNORD113-1 siRNA (Figure 3A). Cells treated with empty vector or scrambled siRNA showed no significant differences in SNORD113-1 expression.Three days following transfection, the viability of cells transfected with p3.1-SNORD113-1 decreased 28.7 ~ 30.2% compared with cells transfected with empty vector or non-transfected cells. However, the viability of cells transfected with SNORD113-1 siRNA increased by 31.5 ~ 32.3% (Figure 3B). These results indicate that SNORD113-1 suppressed HCC cell growth. To validate the suppression of SNORD113-1 on cell growth, we performed colony formation assays in HepG2 and Huh7 transfected with p3.1-SNORD113-1 or transfected with SNORD113-1 siRNA. As shown in Figure 3C, HepG2 and Huh7 cells transfected with p3.1-SNORD113-1 formed much fewer and smaller colonies (135 or 351 colonies) compared with empty vector transfected (491 or 892 colonies) and non-tranfected cells (483 or 857 colonies). In contrast, cells transfected with SNORD113-1 siRNA formed more numerous and larger colonies (618 or 1043 colonies). To further confirm the above findings, a xenograft mouse model was used. As shown in Figure 3D, tumors generated by injection with p3.1-SNORD113-1 transfected HepG2 cells were significant smaller after 5 weeks, compared with the scramble siRNA transfected or non-transfected HepG2 cell groups. However, tumors generated from SNORD113-1 siRNA transfected HepG2 cells were significant larger 4 and 5 weeks after injection. These results indicate that introduction of SNORD113-1 significantly suppressed tumorigenesis in a xenograft nude mouse model.Cell cycle analysis showed that the percentages of p3.1-SNORD113-1 transfected HepG2 cells in the S phase were 8.85% more than that of empty vector transfected or non-transfected cells, with a parallel 7.72% decrease of cells in the G2-M phase (Figure 3E). In SNORD113-1 siRNA transfected cells, the percentage of cells in the S phase was 6.93% less than that of scramble siRNA transfected or non-transfected cells, which corresponded with a 6.99% increase in cells in the G2-M phase (Figure 3E). There were no significant differences in the total duration of the cell-cycle between p3.1-SNORD113-1 or SNORD113-1 siRNA transfected and non-transfected cells (data not shown). Similar results were observed in Huh7 cells. In addition, in both p3.1-SNORD113-1 and SNORD113-1 siRNA transfected cells, the percentages of apoptotic cells were similar to that of the control group (Figure 3F). Together, these results suggest that SNORD113-1 could suppress HCC cell proliferation by inducing cell cycle arrest rather than apoptosis.To further examine the effects of SNORD113-1 on HCC tumorigenesis, the migration of HCC cells in transwell culture chambers was investigated. As shown in Figure 3G, the migration rates of p3.1-SNORD113-1 or SNORD113-1 siRNA transfected HepG2 cells were similar. In addition, Matrigel invasion assays were carried out to determine the effects of SNORD113-1 on the invasive behavior of HCC cells, however, no significant differences were observed (Figure 3G). These results suggested that SNORD113-1 had no effect on the migration and invasion of HCC cells.

### MAPK/ERK and TGF-β pathway were involved in SNORD113-1 effects

**Figure 4:**
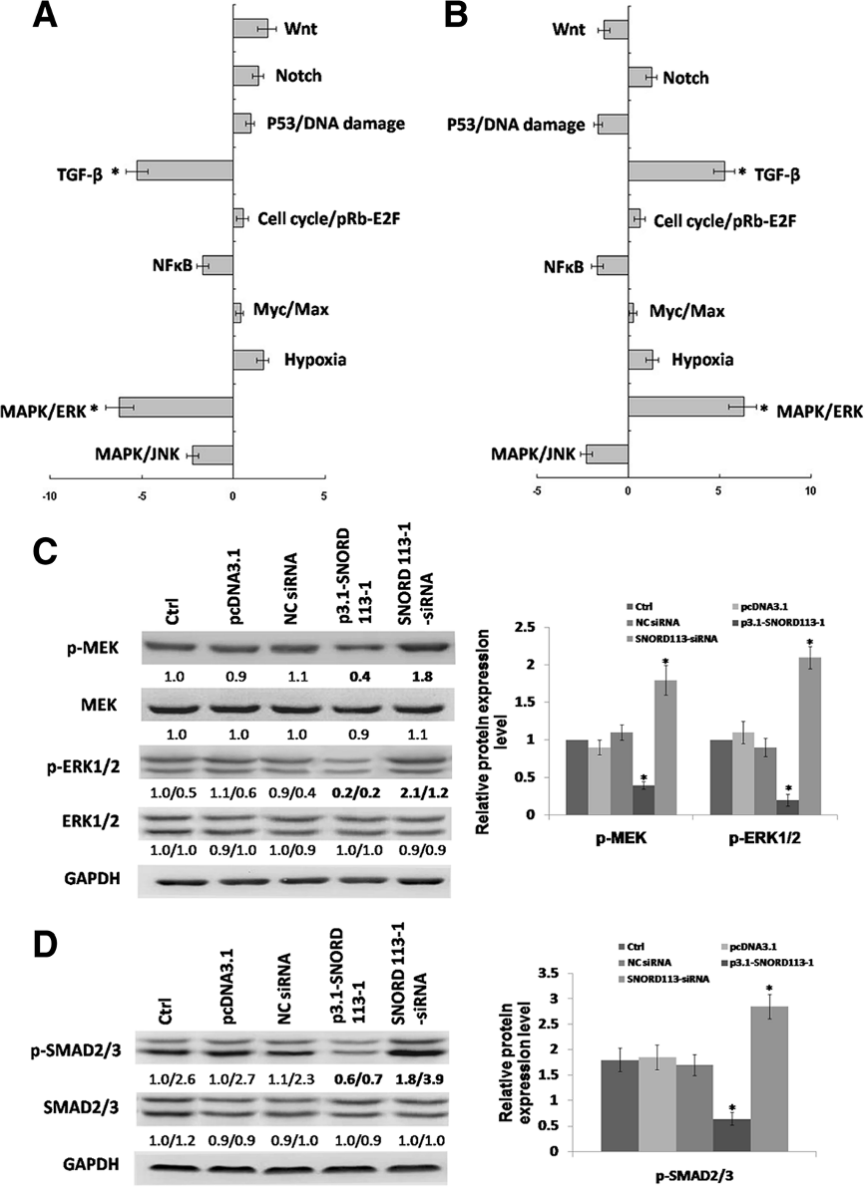
**MAPK/ERK and TGF-β pathway were involved in SNORD113-1 effect. A**, **B**. The relative reporter expression in HepG2 transfected with p3.1-SNORD113-1 **(A)** or SNORD113-1 siRNA **(B)** determined using Cignal Finder 10 Cancer Pathway Reporter Assays. HepG2 cells transfected with p3.1-SNORD113-1 or SNORD113-1 siRNA were seeded onto 96-well plates containing luciferase reporters to 10 common cancer pathways along with FuGene HD transfection reagent. Luciferase activity of the cells was measured using the dual luciferase assay system. Relative firefly luciferase activity was calculated and normalized to the constitutively expressed *Renilla* luciferase. **C**, **D**. The effect of SNORD113-1 on MAPK/ERK pathway **(C)** or TGF-β pathway **(D)**. The expression and phosphorylation of key molecules of the pathways were detected using immunoblotting. GAPDH was used as an internal control.

In order to identify SNORD113-1 target genes, the global mRNA expression in HepG2 cells transfected with either p3.1-SNORD113-1 or SNORD113-1 siRNA was analyzed with Human Gene Expression Arrays. Unexpectedly, SNORD113-1 had little effect on global gene expression; only a very small number of genes that demonstrated significant changes in expression levels (fold change ≤ -0.5 or ≥ +2, P-value ≤ 0.05) after SNORD113-1 overexpression or knockdown were identified (data not shown). These results suggest that SNORD113-1 might exert inhibitory effects through a novel indirect mechanism.To investigate the mechanism of SNORD113-1 suppression of tumor growth in HCCs, 10 major cancer-related pathways were analyzed in p3.1-SNORD113-1 or SNORD113-1 siRNA transfected HepG2 cells using a dual-luciferase reporter system (Promega). Figure 4A shows the relative reporter expression in HepG2 cells transfected with p3.1-SNORD113-1 normalized to that of cells transfected with pcDNA3.1. The most affected pathways were transforming growth factor-β (TGF-β) and mitogen-activated protein kinase (MAPK)/extracellular signal-regulated kinase (ERK). Figure 4B shows the relative reporter expression in HepG2 cells transfected with SNORD113-1 siRNA normalized to that of cells transfected with NC siRNA. The most affected pathways were also TGF-β and MAPK/ERK. The downregulation of the MAPK/ERK and TGF-β pathways in cells overexpressing SNORD113-1 were consistent with the upregulation of the MAPK/ERK and TGF-β pathways in SNORD113-1 knockdown cells.To verify these findings, the key molecules in the MAPK/ERK and TGF-β pathways were detected by immunoblotting. SNORD113-1 overexpression significantly decreased phosphorylation of MEK and ERK1/2 (Figure 4C), whereas total ERK and total MEK expression were not significantly altered (Figure 4C). Similarly, upon examination of the TGF-β pathway, phosphorylation of SMAD2/3 was decreased by SNORD113-1 overexpression (Figure 4D). These results were reversed under conditions of SNORD113-1 knockdown. These results indicate that both the MAPK/ERK and TGF-β pathways may be involved in SNORD113-1 suppression of tumorigenesis.

## Discussion

Among the upregulated genes, PGC, AFP, AKR1B10 and GPC3 showed more than 30-fold increased expression in the HCC tumor group. AFP is the only serum biomarker that has widely been used in the diagnosis of HCC [11]; GPC3 is also a potential reliable biomarker and therapeutic target in HCC [12]; PGC is overexpressed in HCC [13]; AKR1B10 is a valuable novel biomarker candidate for staging of HCC [14]. In addition, other genes, including secreted phosphoprotein 1 (SPP1, osteopontin), fatty acid desaturase 2 (FADS2) and serine peptidase inhibitor kazal type 1(SPINK1), also have been reported to be associated with HCC progression [15–17]. Among the downregulated genes, TAT, JDP2, HSD17B13, CYP2B6 and PCK1 showed more than 30-fold decreased expression in HCC tumors, and all of these genes have been reported to be associated with HCC progression previously, except HSD17B13 [18–21]. The identification of these well-known HCC biomarkers suggested that the results of the array were reliable. In order to identify novel molecular and potential biomarkers for HCC tumorigenesis, downregulated snoRNAs were chosen for this study.

With regard to a link between snoRNA and tumorigenesis, Dong et al. reported that U50 undergoes frequent genomic heterozygous deletions and transcriptional downregulation, and U50 overexpression reduces the colony-forming ability in both prostate and breast cancer cells [22, 23]. Horsthemke et al. reported that a diverse number of snoRNAs are differentially expressed in non-small cell lung cancer with respect to the corresponding matched tissue [24]. More recently, Mei et al. reported that SNORA42 expression in non-small cell lung cancer cells increased colony formation in culture and tumorigenesis [7]. Together, these findings suggest a possible role of snoRNAs in oncogenesis. In the present study, SNORD113-1 exhibited a strong ability to inhibit cell growth and proliferation in human HCC cells. In addition, the results from a xenograft animal model provided the direct evidence that SNORD113-1 can regulate HCC tumor cell growth and that the loss of SNORD113-1 gene function may be directly associated with tumor development. The activity of SNORD113-1 caused growth suppression. In addition, SNORD113-1 was expressed in normal liver tissues but absent in paired HCC tissues, suggesting that it may play an important role in the maintenance of normal cell growth. These exciting findings suggest that SNORD113-1 may play a role as a tumor suppressor gene. However, the sequence of SNORD113-1 was cloned into a plasmid and overexpressed in cells, and the correct processing of the sequence into the mature SNORD113-1 requires further confirmation.

The SNORD113-1 gene is located at chromosome 14q32 within an intron of the small nucleolar RNA host gene 23 (SNHG23, Gene ID: 100507242) together with many miRNAs and other C/D snoRNAs. Interestingly, genomic alterations in the 14q32 locus have been observed in HCC and colorectal carcinomas [25, 26]. Loss of heterozygosity (LOH) is considered to be an indicator of the presence of a tumor suppressor gene (TSG), whereas inactivation at the locus contributes to the development and/or progression of tumors [27]. In the present study, LOH of the SNORD113-1 gene was found in only 11.1% (4/36) HCC tumor tissues (data not shown), which was less than the 43.6% LOH reported previously [25, 26]. Zhang et al. suggested that allelic loss at the MEG3 locus was more prevalent in higher grade tumors [28], and this discrepancy in our results compared to those previously reported might be due to differences in the grades in the HCC tumors examined in each study.

In most cases, the levels of the intron encoded snoRNA genes seem to be determined by the transcription of their host genes [29]; thus the transcription of those snoRNA might be synchronized to their host genes. The intergenic snoRNA genes are independently transcribed from their own promoters by RNA polymerase II (Pol II) [30]. Generally, the 5′UTR lengths of protein-coding genes are within 50 bps to 100 bps. In contrast, the distances between intergenic miRNA transcription start sites (TSS) and their precursor’s TSS significantly fluctuate from less than 100 bps to 50 kb [31, 32]. Using CpG island prediction software, we did not identify any CpG islands within the 3000 bp upstream region of the SNORD113-1 gene. The region from 2000 bp upstream of MEG8 to the 0 bp upstream region of SNORD113-1 was analyzed with the CpG island prediction software. Sequence information showed that a region (fragment 1) located approximately 12 kb upstream of SNORD113-1 gene was rich in CpG dinucleotides, suggesting that this region was a putative promoter region of SNORD113-1. The results of sodium bisulfite treatment demonstrated that the SNORD113-1 fragment 1 in HCC tumors contained significantly higher numbers of methylated CpGs than those in non-tumor tissues. Studies have also shown that epigenetic silencing of snoRNAs with tumor suppressor activities by CpG island hypermethylation is a common feature of human cancers. Ferreira et al. reported that 43.5% (20/46) of snoRNAs were fully methylated in the colorectal cancer cell line hcT-116, which suggests that CpG island hypermethylation is associated with the transcriptional silencing of their respective snoRNAs [33]. The results of DNA methylation microarray platform demonstrated that the observed hypermethylation of snoRNAs was a common feature of various tumor types [33]. MEG3 has been shown to be methylated in human renal cell carcinoma tissues and cell lines, as well as in pheochromocytomas and human pituitary tumors [34–36], and promoter hypermethylation downregulated MEG3 expression and promotes oncogenesis [36]. Thus, the fragment 1 was the most likely promoter region for SNORD113-1.

Most noncoding RNA promoters are computationally predicted without experimental validation. Until now, only a few of the promoters predicted using chromatin signatures have been confirmed by promoter reporter assays [37, 38]. In the present study, fragment 1 clearly demonstrated strong promoter activity in activation of reporter gene expression. In addition, the expression of SNORD113-1 in HepG2 cells induced by a demethylation reagent indicates that methylation is related to SNORD113-1 gene transcription. Taken together, these results suggest that hypermethylation of fragment 1 is an important mechanism associated with the loss of SNORD113-1 expression in human HCC tumors. However, it has been shown that non-CpG promoters demonstrated the highest levels of prediction inaccuracy [39], and it is currently unclear whether there are non-CpG promoters within the 8000 bp region from fragment 1 to fragment.

Two distinct, differentially methylated regions (DMR) are present in the Dlk1-Dio3 region and have separate functional properties: a primary, germline-derived Dlk1-MEG3 intergenic DMR (IG-DMR) and a secondary, post-fertilization-derived MEG3-DMR [10]. Seitz H, et al. concluded that the miRNA genes tested in Dlk1-Dio3 region exhibited a tissue-specific expression pattern, and they were only expressed from the maternally inherited allele with imprinted expression controlled by a differentially methylated region (DMR) located ∼ 200 kb away [40]. Manodoro F, et al. also reported the methylation of DMR was associated with acute promyelocytic leukemia [41]. However, our findings are different from these previous studies. In the present study, we also examined the methylation status of DMR and the expression of genes, snoRNAs and miRNAs in the Dlk1-Dio3 region of our HCC tumors compared with adjacent non-tumor tissues. No significant differences in methylation within the IG-DMR were found between HCC tumors and adjacent non-tumor tissues (data not shown). In addition, the expressions of Dlk1, SNORD113-3, miR-370 and miR-485, and others were significantly downregulated, but the expressions of Dio3, miR-127, miR-136, miR-154 and miR-544, and others demonstrated no significant changes (Additional file 2: Table S3). These results revealed that the expression of SNORD113 and SNORD114 clusters and miRNAs which are located behind the putative promoter region (F1 in this study) have similar expression patterns, suggesting that they might share the same promoter. However, differential expression patterns of some miRNAs in the Dlk1-Dio3 region suggest that there are additional regulatory mechanisms, such as post-transcriptional modifications.

The sequences of snoRNAs are responsible for targeting the assembled snoRNPs to a specific target [42]. SnoRNAs and snoRNPs are likely to contribute to tumorigenesis through effects on ribosomes and protein translation, given that translation is often perturbed in cancer cells. However, snoRNAs might also be involved in the regulation of gene expression by giving rise to other regulatory RNA species, such as miRNAs [43]. To further examine whether SNORD113-1 could affect gene expression and explore its possible functions, HepG2 cells transfected with p3.1-SNORD113-1 or SNORD113-1 siRNA were analyzed by mRNA transcript arrays. SNORD113-1 showed little effects on global gene expression in the mRNA transcript arrays, which is consistent with previous reports examining other small nucleolar RNAs [44]. Furthermore, MAPK/ERK and TGF-β pathways were found altered in response to changes in SNORD113-1 expression levels. Immunoblotting analyses showed that phosphorylated MEK and ERK compared to total ERK, as well as phosphorylated SMAD2/3 compared to total SMAD2/3 and SMAD4, were increased both in SNORD113-1 siRNA transfected HepG2 cells and in HCC tumor tissues. SNORD113-1 had few effects on mRNA levels but significant effects on protein phosphorylation levels suggesting that snoRNAs may act through indirect mechanisms on these targets. The MAPK/ERK signaling pathway is involved in diverse cellular processes such as cell survival, differentiation and proliferation [45], and overexpression of members of this pathway was found to be correlated to HCC [46]. Similarly, the TGF-β pathway also regulates cell proliferation, differentiation and adhesion [47]. These two pathways have been reported to play significant roles in HCC tumorigenesis [48]. These results suggest MAPK/ERK and TGF-β pathways are most likely involved in SNORD113-1 suppression of HCC tumorigenesis.

In the present study, downregulation of SNORD113-1 was frequently observed in HCC tumors but rarely present in non-tumor tissues. Importantly, SNORD113-1 expression was correlated with disease-free survival of HCC patients. These results obtained from clinical specimens provide evidence to support the hypothesis that decreased expression of SNORD113-1 contributes to HCC development and progression. In addition, overexpression of SNORD113-1 could inhibit cell viability and proliferation of cancer cells, and thus have an important role in the development of HCC. Therefore, SNORD113-1 may present not only a useful molecular marker for selecting patients with poor prognosis to receive more personalized therapy, but also a potential therapeutic target for HCC. Nevertheless, validating its prognostic value in a large population and developing novel strategies for improving treatment efficiencies of HCC are needed.

## Conclusions

In conclusion, the results from these studies demonstrated that SNORD113-1 suppresses HCC tumorigenesis in MAPK/ERK and TGF-β pathway-dependent mechanisms.

## Methods

### Cell lines and tissue specimens

Human embryonic kidney cell line Hek293T and human HCC cell lines HepG2 and Huh7 were cultured in DMEM medium supplemented with 10% FBS (Life Technology, Carlsbad, CA) at 37°C in a humidified atmosphere containing 5% CO_2_.

112 pairs of human hepatitis B virus (HBV) associated HCC and adjacent non-tumor tissues were obtained from surgical specimens immediately after resection from patients undergoing primary surgical treatment of HCC in the Eastern Hepatobiliary Surgery Hospital, Shanghai, China. The samples were frozen in liquid nitrogen and stored at -80°C for use in experiments. Among these samples, three pairs were used for mRNA array analyses (Table  1A) and all of them were used for quantitative real time PCR (qRT-PCR) analyses (Table  1B). Clinical and pathological information was extracted from the patients’ medical charts and pathology reports (Table  1). Written consent for tissue donation (for research purposes) was obtained from the patients prior to tissue collection and the protocol was approved by the Institutional Review Board of Eastern Hepatobiliary Surgery Hospital and Second Military Medical University.

### Total RNA extraction and gene expression profiling

Total RNA was isolated using TRI Reagent combined with the RNeasy Tissue kit protocol (Qiagen, Valencia, CA) according to the manufacturer’s recommendations. The RNA concentrations and the A260 nm/A280 nm ratios were assessed with a multi-plate reader (Synergy 2; BioTek, Winooski, VT). The 28S/18S ratio and the RNA integrity number were assessed with a Bioanalyzer 2100 (Agilent Technologies, Wilmington, DE). An A260 nm/A280 nm ratio of 1.9, a 28S/18S ratio of 1.8, and an RNA integrity number of 5 were minimum requirements for inclusion in expression analysis.

To identify differentially expressed genes in HCC tumor tissues, The Glue Grant Human Transcriptome Arrays (Affymetrix, Santa Clara, CA) were performed on 3 pairs of HCC and adjacent non-tumor tissues (Table 1A) according to the manufacturer’s protocol by Gminix Corp. (Shanghai, China). For SNORD113-1 related gene identification, the global mRNA expression of HepG2 cells transfected with p3.1-SNORD113-1, SNORD113-1 siRNA, empty vector, scramble siRNA or untransfected cells were analyzed with the PrimeView™ Human Gene Expression Array (Affymetrix) according to the manufacturer’s protocol by Shanghai Biotechnology Corp. (Shanghai, China).

A total of 750 ng of labeled complementary RNAs were hybridized to arrays and then imaged using Affymetrix Fluidics Station FS450 and scanned with GeneChip Scanner 3000 7G according to manufacturer’s instructions. Raw signals of the arrays were processed using Affymetrix Power Tools. Data quality was assessed based on the positive and negative control probes on each array as well as by inspection of the distributions of probe intensities. Data was normalized using the quantile normalization method. A moderated t-test implemented in the limma library of bioconductor was applied to test differential expression, and a false discovery rate (FDR) adjustment of the p-value was performed to correct for multiple testing. Probes were considered significantly different if the adjusted p-value was less than 0.05 and the fold change difference between groups was at least 2.

### Quantitative real time PCR

Quantitative real time PCR (qRT-PCR) analysis of SNORD113-1 expression was carried out according to the manufacturer’s protocol. Briefly, total RNA was extracted using TRIzol Reagent (Invitrogen) from HCC tissues or cell lines and was used to synthesize cDNAs with SNORD113-1 specific reverse primers. The reactions were incubated for 30 min at 16°C, 30 min at 42°C, 5 min at 85°C, and then held at 4°C. The cDNA product was used for qRT-PCR analysis directly with primers for SNORD113-1 (Additional file 3: Table S1). Reactions were incubated at 95°C for 5 min, followed by 40 cycles at 95°C for 15 sec and 60°C for 1 min. PCR reactions were run on a StepOne Plus real time PCR machine (Applied Biosystems) and the data were analyzed using SDS v2.3 software. Glyceraldehyde-3-phosphate dehydrogenase (GAPDH) and U6 were used as reference controls for normalization. For 5-aza-2′-deoxycytidine treatment**,** HepG2 cells were seeded into 60 × 15-mm tissue culture dishes and cultured in DMEM containing 5 μmol/L 5-aza-2′-deoxycytidine (5-Ad, Sigma-Aldrich, St. Louis, MO) for 48 hours. Cells cultured in the absence of 5-Ad were used as a negative control. SNORD113-1 mRNA expression was determined using qRT-PCR as described above. The PCR products were also separated by 1.5% agarose gel electrophoresis, visualized and analyzed by the Tanon UV-2000 (Tanon, Shanghai, China).

### Plasmid constructions, transfection and luciferase reporter assay

The synthesized SNORD113-1 (AAAGTGAGTGATGAATAGTTCTGTGGCATA TGAATCATTAATTTTGATTAAACCCTAAACTCTGAAGTCC, Genebank: NR_003229.1, by Genery, Shanghai, China) was cloned into pcDNA3.1 vector (Life Technology). The constructs were confirmed by sequencing and termed as p3.1-SNORD113-1. The expression of SNORD113-1 was confirmed by qRT-PCR.

Transfection was carried out using FuGene HD transfection reagent (Roche, Indianapolis, IN) following the manufacturer’s protocol. In brief, 2 × 10^4^ HepG2 and Hek293T cells in 24-well plates were transfected with the indicated plasmids, specific or scramble siRNA (GenePharma, Shanghai, China), and collected 48 hours after transfection for assays.

The fragments of the putative promoter region (F1, Figure 2B) and 3000 bp upstream of the gene of SNORD113-1 (which was divided to three fragments of 1000 bp) (F2, F3 and F4, Figure 2B) were amplified with primers (Additional file 3: Table S1) and cloned into a promoter-less luciferase reporter vector pGL3-Basic (Promega Corp. Madison, WI), termed as pGL3-F1, pGL3-F2, pGL3-F3 and pGL3-F4. HEK293T cells were seeded on a 24-well plate in triplicate and transfected with pGL3-F1, pGL3-F2, pGL3-F3, pGL3-F4 or empty vector pGL3 using FuGene HD transfection reagent. The pRL-TK was also transfected as a control for normalization. Cells were collected 48 hours after transfection, and luciferase activity was measured using a dual-luciferase reporter assay kit (Promega Corp.) and measured using a multi-plate reader (Synergy 2, BioTek).

### Sodium bisulfite sequencing

CpG islands were analyzed using CpG island searcher (http://www.cpgislands.com/), CoreBoost_HM (http://rulai.cshl.edu/tools/CoreBoost_HM/) and MethPrimer (http://www.urogene.org/methprimer/index1.html). Genomic DNA from HCC tumor tissues or adjacent non-tumor tissues was extracted using DNeasy Tissue Kit (QIAGEN, Valencia, CA). One microgram of genomic DNA was treated with sodium bisulfite using the CpGenome DNA Modification Kit (Serologicals Corp., Norcross, GA) according to the manufacturer’s protocol. Hotstart PCRs were performed with the primers (Additional file 3: Table S1, bisulfit-1 and bisulfit-2) under the following conditions: 95°C for 15 min, 94°C for 30 sec, 64°C for 30 sec, and 72°C for 2 min for 40 cycles, and 72°C for 10 min. PCR products were subcloned, and five constructs representing each region from each sample were randomly selected for sequence analysis. DNA methylation data were analyzed and visualized using BiQ Analyzer (http://biq-analyzer.bioinf.mpi-sb.mpg.de/).

### Cell viability, colony formation assay, cell cycle and apoptosis analysis

Twenty four hours following transfection, 1000 transfected HepG2 or Huh7 cells were plated on a fresh 96-well plate in triplicate and maintained in DMEM containing 10% FBS for 5 days to assess cellular viability. Cells were tested for proliferation every 24 hours using Cell Titer-Blue cell viability assay (Promega Corp.) according to the manufacturer’s instructions and the fluorescence values were recorded using a multi-plate reader (Synergy 2, BioTek). For colony formation assays, 2000 transfected HepG2 or Huh7 were plated on a fresh 6-well plate in triplicate and maintained in DMEM containing 10% FBS for 2 weeks. Cell colonies were fixed with 20% methanol and stained with 0.1% coomassie brilliant blue R250 at room temperature for 15 min. The colonies were counted using an ELIspot Bioreader 5000 (BIO-SYS, Karben, GE).

Forty eight hours following transfection, 1 × 10^5^ transfected HepG2 or Huh7 cells were harvested, washed once in phosphate buffer saline (PBS), and fixed in 70% ethanol at 4°C overnight. Staining for DNA content was performed with 50 mg/mL propidium iodide and 1 mg/mL RNase A at room temperature for 30 minutes. Populations in G0-G1, S, and G2-M phase were measured using a Cell Lab Quanta SC flow cytometer (Beckman Coulter, Fullerton, CA), and the data were analyzed using FlowJo v7.6 Software. For apoptosis analysis, cells were incubated with FITC-Annexin V (Promega Corp.) for 15 minutes at 4°C in the dark, according to the manufacturer’s instructions and measured with the same system. In addition, caspase 3/7 activities of transfected HepG2 or Huh7 cells were also measured with the Caspase-Glo 3/7 Assay (Promega Corp.) according to manufacturer’s instructions and recorded with a multi-plate reader (Synergy 2, BioTek).

### Tumorigenicity assay in xenograft nude mice

Male BALB/c nude mice (5 to 6 weeks of age) were obtained from Shanghai Experimental Animal Center (Shanghai, China). Animal handling and experimental procedures were approved by the Animal Experiments Ethics Committee of Second Military Medical University. For the *in vivo* tumorigenicity assays, all pyrimidine nucleotides in the SNORD113-1 siRNA or scramble siRNA were substituted with their 2′-O-methyl analogues to improve RNA stability. HepG2 cells (1 × 10^6^) transfected with p3.1-SNORD113-1 or SNORD113-1 siRNA were suspended in 100 μL PBS and then injected into the left side of the posterior flank of 6 BALB/c nude mice each. Scramble siRNA or empty vector transfected HepG2 cells (1 × 10^6^) were injected subcutaneously into the right side of same 12 mice. Tumor growth was examined daily, and the tumor volumes were calculated every week using the formula for hemi-ellipsoids: V = length (cm) × width (cm) × height (cm) × 0.5236. After 5 weeks, the mice were sacrificed and the tumors were dissected and imaged.

### Cell migration and invasion assays

For the cell migration assay, 2 × 10^5^ HepG2 cells transfected with p3.1-SNORD113-1 or SNORD113-1 siRNA were seeded in the upper chamber of transwell units (Corning, NY, USA) with 8 μm pore size polycarbonate filters under serum-free conditions. The lower chamber was filled with 500 μL DMEM containing 10% FBS. After incubation for 24 h, cells on the upper surface of the filter were completely removed by wiping with a cotton swab. Then the filters were fixed with 4% paraformaldehyde and stained with 0.1% coomassie brilliant blue R250 for 20 min. Cells that migrated through the pores to the lower surface of the filter were counted and analyzed with a digital microscope system (IX81; Olympus). Triplicate samples were acquired, and the data were expressed as the average cell number of 5 fields. For the cell invasion assays, a protocol similar to the cell migration assay was used, except that the transwell units were pre-coated with 200 μg/ml Matrigel (BD Biosciences, San Jose, CA, USA) and incubated overnight. Cells that invaded through the Matrigel and reached the lower surface of the filter were counted.

### Immunoblotting

Protein extracts from HCC tissues or HepG2 cells were prepared using a modified RIPA buffer with 0.5% sodium dodecyl sulfate (SDS) in the presence of proteinase inhibitor cocktail (Complete mini, Roche, Indianapolis, IN, USA). Fifty micrograms of protein from HCC tissues and their adjacent non-tumor tissues were electrophoresed in 10% SDS-PAGE mini gels and transferred onto PVDF membranes (Immobilon P^-SQ^, Millipore, Billerica, MA, USA). After blocking with 5% nonfat milk, the membranes were incubated with primary antibodies at 4°C overnight, followed by incubation with HRP-conjugated goat anti-rabbit or goat anti-mouse antibody (1:10000 dilution, KPL, Gaithersburg, MA,USA) for 1 hour at room temperature. Signals were developed with Super Signal West Pico chemiluminescent substrate (Pierce, Rockford, Il, USA), visualized using the Gene Gnome HR Image Capture System (Syngene, Frederick, MD, USA), and analyzed with Gene tools (Syngene). The primary antibodies were: MEK, p-MEK, ERK, p-ERK, SMAD2/3, p-SMAD2/3 (1:1000 dilution, Cell Signaling Technology, Danvers, MA, USA), and GAPDH (1:5000 dilution, Epitomics Inc., Burlingame, CA, USA).

### Cancer pathway reporter assays

HepG2 cells transfected with p3.1-SNORD113-1 or SNORD113-1 siRNA were examined using Cignal Finder 10 Cancer Pathway Reporter Assays (Qiagen Inc., Valencia, CA, USA) according to the manufacturer’s protocol. In brief, cells were seeded onto 96-well plates containing luciferase reporters from 10 common cancer pathways along with FuGene HD transfection reagent. Forty eight hours later, the luciferase activities of cells were measured using the Dual Luciferase Assay system (Promega). Relative *firefly* luciferase activity was calculated and normalized to the constitutively expressed *Renilla* luciferase.

### Statistical analysis

All experiments were performed at least three times, and data are presented as mean ± SD. Comparisons were made by using a two-tailed t test or one-way ANOVA for experiments with more than two subgroups. Correlation analysis was made by using Spearman correlation coefficient. Association of SNORD113-1 expression with cancer specific survival rate was analyzed using the Kaplan-Meier method.

## Electronic supplementary material

Additional file 1: Table S2: The CpG islands predicted by three softwares. (DOC 38 KB)

Additional file 2: Table S3: The expression of genes and miRNAs, snoRNAs in Dlk1-Dio3 region*. (DOC 34 KB)

Additional file 3: Table S1: Primers used in this study. (DOC 34 KB)
