# Supplementary material for: Small nucleolar RNA 113–1 suppresses tumorigenesis in hepatocellular carcinoma
Source: Mol Cancer. 2014 Sep 14;13:216. doi: 10.1186/1476-4598-13-216 (PMC4169825; doi:10.1186/1476-4598-13-216)
Supplement: Supplementary file 1 — Additional file 1: Table S2: The CpG islands predicted by three softwares. (DOC 38 KB) [file 12943_2014_1411_MOESM1_ESM.doc]

**Table S2. The CpG islands predicted by three softwares.**

| **Chromosome 14**  **(82359108~82391159)** | **CpG Island Searcher** |  | **CoreBoost_HM** |  | **MethPrimer** |  |
| --- | --- | --- | --- | --- | --- | --- |
| **CpG islands** | **Region** | **Length** | **Region** | **Length** | **Region** | **Length** |
| **1** | / | / | 82359910~82360274 | 364bp | 82359936~82360067 | 131bp |
| **2** | / | / | 82363133~82363378 | 245bp | 82363136~82363236 | 101bp |
| **3** | / | / | 82366622~82366916 | 294bp | 82366682~82366792 | 111bp |
| **4** | **82370922~82371234** | 313bp | **82370762~82371124** | 362bp | **82370922~82371234** | 313bp |
| **5** | / | / | / | / | 82375974~82376078 | 105bp |
| **6** | / | / | 82377261~82377541 | 280bp | 82377332~82377498 | 166bp |
| **7** | / | / | 82385580~52385826 | 246bp | / | / |
