# Supplementary material for: Small nucleolar RNA 113–1 suppresses tumorigenesis in hepatocellular carcinoma
Source: Mol Cancer. 2014 Sep 14;13:216. doi: 10.1186/1476-4598-13-216 (PMC4169825; doi:10.1186/1476-4598-13-216)
Supplement: Supplementary file 2 — Additional file 2: Table S3: The expression of genes and miRNAs, snoRNAs in Dlk1-Dio3 region*. (DOC 34 KB) [file 12943_2014_1411_MOESM2_ESM.doc]

**Table S3. The expression of genes and miRNAs, snoRNAs in Dlk1-Dio3 region*.**

| **Genes** | Percentage of downregulation in HCC tumor tissue/nontumor (n=36) |
| --- | --- |
| **Downregulation**  Dlk1  SNORD113-3 | 24(66.7%)  21(58.3%) |
| SNORD113-5  SNORD113-6 | 20(55.6%)  21(58.3%) |
| SNORD114-1  SNORD114-2  SNORD114-6 | 24(66.7%)  21(58.3%)  23(63.9%) |
| SNORD114-11 | 21(58.3%) |
| SNORD114-17 | 23(63.9%) |
| miR-337  miR-370  miR-379  miR-485  miR-487  miR-495  miR-411  miR-655  **No significant change** | 26(72.2%)  24(66.7%)  21(58.3%)  24(66.7%)  21(58.3%)  23(63.9%)  21(58.3%)  23(63.9%) |
| SNORD112-1  Dio3  miR-127  miR-136  miR-154  miR-323 | 6(16.7%)  8(22.2%)  3(8.3%)  5(13.9%)  4(11.1%)  3(8.3%) |
| miR-329  miR-380  miR-376  miR-494  miR-496  miR-544 | 3(8.3%)  4(11.1%)  6(16.7%)  4(11.1%)  5(13.9%)  3(8.3%) |

*: The expression of genes and miRNAs, snoRNAs was determined by qRT-PCR.
