# Supplementary material for: Small nucleolar RNA 113–1 suppresses tumorigenesis in hepatocellular carcinoma
Source: Mol Cancer. 2014 Sep 14;13:216. doi: 10.1186/1476-4598-13-216 (PMC4169825; doi:10.1186/1476-4598-13-216)
Supplement: Supplementary file 3 — Additional file 3: Table S1: Primers used in this study. (DOC 34 KB) [file 12943_2014_1411_MOESM3_ESM.doc]

**Table S1. Primers used in this study.**

| **Genes** | **Forward primer** | **Reverse primer** |
| --- | --- | --- |
| SNORD113-1 | AGTGAGTGATGAATAGTTCTGTG | GACTTCAGAGTTTAGGGTTTAATC |
| F1 | TGGAGGGAGAAGGGAGAGGTGTG | CCCCACTCTTCCCCTATCTCTG |
| F2 | TAGTTTTGGGGGAACAGGTG | TGCAAAAACAAGTGCAACAA |
| F3 | CCAATTATCCCAGCAGCATT | GAAAACTCCCAAACCTGTGC |
| F4 | TTTTTAGGGCACAGGTTTGG | TGTTTCCCACAGAGCTGGTAG |
| Bisulfit-1 | GGGCTGCCTGGGCCACATTTC | TGTGAGGGAGGGGCGCAACC |
| Bisulfit-2 | GGGCTGCAGGTTGCGCCCCTC | CTCCTCCCCCACTCTTCTCTTTC |
| U6 | CTCGCTTCGGCAGCACA | AACGCTTCACGAATTTGCGT |
| GAPDH | TGCACCACCAACTGCTTAG | AGTAGAGGCAGGGATGATGTTC |
